# Peripheral immune cell profiling of double-hit lymphoma by mass cytometry

**DOI:** 10.1186/s12885-023-10657-0

**Published:** 2023-02-23

**Authors:** Tao Lei, Gongqiang Wu, Yongjin Xu, Weihao Zhuang, Jialiang Lu, Shuiyun Han, Yuxin Zhuang, Xiaowu Dong, Haiyan Yang

**Affiliations:** 1 Department of Lymphoma, Institute of Basic Medicine and Cancer (IBMC), The Cancer Hospital of the University of Chinese Academy of Sciences (Zhejiang Cancer Hospital), Chinese Academy of Sciences, Hangzhou, P. R. China; 2 Department of Hematology, Dongyang People’s Hospital, Dongyang Hospital Affiliated to Wenzhou Medical University, Dongyang, Zhejiang P. R. China; 3 Hangzhou Institute of Innovative Medicine, Institute of Drug Discovery and Design, College of Pharmaceutical Sciences, Zhejiang University, Hangzhou, P. R. China; 4 Innovation Institute for Artificial Intelligence in Medicine of Zhejiang University, Hangzhou, P. R. China; 5 Cancer Center, Zhejiang University, Hangzhou, P. R. China

**Keywords:** Double-hit Lymphoma, Peripheral blood, Immune cells, CyTOF, CD38, Heterogeneity

## Abstract

**Background:**

Double-hit or Triple-hit lymphoma (DHL/THL) is a subset of high-grade B cell lymphoma harboring rearrangements of *MYC* and *BCL2* and/or *BCL6*, and usually associate with aggressive profile, while current therapies tend to provide poor clinical outcomes and eventually relapsed. Further explorations of DHL at cellular and molecular levels are in demand to offer guidance for clinical activity.

**Methods:**

We collected the peripheral blood of DHL patients and diffused large B cell lymphoma (DLBCL) patients from single institute and converted them into PBMC samples. Mass cytometry was then performed to characterize these samples by 42 antibody markers with samples of healthy people as control. We divided the immune cell subtypes based on the expression profile of surface antigens, and the proportion of each cell subtype was also analyzed. By comparing the data of the DLBCL group and the healthy group, we figured out the distinguished immune cell subtypes of DHL patients according to their abundance and marker expression level. We further analyzed the heterogeneity of DHL samples by pairwise comparison based on clinical characteristics.

**Results:**

We found double-positive T cells (DPT) cells were in a significantly high percentage in DHL patients, whereas the ratio of double-negative T cells (DNT) was largely reduced in patients. Besides, CD38 was uniquely expressed at a high level on some naïve B cells of DHL patients, which could be a marker for the diagnosis of DHL (distinguishing from DLBCL), or even be a drug target for the treatment of DHL. In addition, we illustrated the heterogeneity of DHL patients in terms of immune cell landscape, and highlighted *TP53* as a major factor that contributes to the heterogeneity of the T cells profile.

**Conclusion:**

Our study demonstrated the distinct peripheral immune cell profile of DHL patients by contrast to DLBCL patients and healthy people, as well as the heterogeneity within the DHL group, which could provide valuable guidance for the diagnosis and treatment of DHL.

**Supplementary Information:**

The online version contains supplementary material available at 10.1186/s12885-023-10657-0.

## Introduction

Double-hit or Triple-hit lymphoma (DHL/THL) is an uncommon subset of B cell non-Hodgkin lymphoma with recurrent translocations involving *MYC/8q24* and *BCL2/18q21* and/or *BCL6/3q27*, according to the 2016 updated WHO classification of lymphoid neoplasias [1]. DHL displays a highly heterogeneous profile with complex genomic alterations, which results in aggressive clinical manifestations and inferior prognosis upon current therapeutic approaches, regardless of targeted therapy or intensive chemotherapy [2, 3].

Despite the increasing clinical interest in DHL and the corresponding precise treatment, few deep explorations of pathogenesis at cellular and molecular level were carried out, and the previous DHL research had mainly focused on genetics and flow cytometric analysis with a limited number of markers measured per single cell [4–6]. On the other hand, the immune system has many important regulatory roles in cancer development and progression, while the immune cell population is of great importance for the function and status of the immune system. Therefore, evaluating immune cell populations of DHL could be a perspective to dig the information behind the clinical phenotype and be possible to uncover immunological mechanisms and identify biomarkers that might aid in precision therapies [7, 8]. Most of the published immune cell profiling of cancers was based on tumor tissue for biomarker identification and analysis [9–11], this is reasonable since the immune cell composition of the tumor site closely reflects the interactions between the cancer cells and immune system. However, acquiring tissue is quite challenging since this approach it is an invasive procedure. Instead, analyses of peripheral blood offer a noninvasive and simpler way to monitor immune cells (peripheral blood mononuclear cells - PBMCs) that ultimately can infiltrate the TME, and therefore may provide valuable information about the status of immune system surrounding the tumor cells [12]. As regards to the approach to characterizing the immune cell of peripheral blood, mass cytometry or CyTOF (cytometry by time of flight) has emerged as a revolutionary technology in single-cell proteomics, enabling a comprehensive understanding of cell phenotype [13]. CyTOF displays tremendous advantages over the conventional flow cytometer, especially the feasibility to measure more than 50 markers per cell in limited samples.

In the present study, we characterized the immune cell profile of a cohort of peripheral blood samples from DHL patients, with blood samples from DLBCL patients and healthy people as the control, and CyTOF was employed with a range of surface markers. This study aimed to understand the discrepancy of peripheral immune cell composition of DHL compared to DLBCL and healthy people and thus identify potential biomarkers for pharmaceutical and clinical utility.

## Materials and methods

### Patient selection

This study was conducted in accordance with the guidelines of the Institutional Review Board of Zhejiang Cancer Hospital. 11 cases of B-cell lymphoma associated with *MYC* and *BCL2* (4 cases), *MYC* and *BCL6* (5 cases), or *MYC*, *BCL2* and *BCL6* rearrangements (2 cases) confirmed by fluorescence in situ hybridization (FISH) studies were identified (Supplementary Table 2). Morphologic, immunophenotypic, and cytogenetic data were reviewed to further confirm the diagnosis and classification according to the 2016 World Health Organization. More clinical information of DHL patients was listed in Table 1. Besides, 4 cases of DLBCL diagnosed at the same institute were identified; pathologic and cytogenetic data were reviewed for all cases to confirm the diagnosis and the lack of rearrangements of *MYC*, *BCL2*, and *BCL6*. In addition, PBMC sample of 7 cases of healthy people were purchased from commercial source (Sailybio).

**Table 1:** Clinical characteristics of DHL patients

Median age (59, range: 42–70, n = 11)	
age < 60 diagnosis	6
age > 60 diagnosis	5
**Sex (n = 11)**	
male	6
female	5
**Disease stage**	
I/II	4
III/IV	7
**B symptoms**	
No	8
Yes	3
**Abdominal lymph nodes**	
No	1
Yes	10
**Marrow involved**	
No	10
Yes	1
**GCB or NGCB**	
GCB	5
NGCB	6
**IPI**	
0–1	3
2	3
3	2
4–5	3
**CNSIPI**	
0–1	3
2	4
4–5	4
***TP53*** **mutation**	
No	7
Yes	3
Undefined	1

### PBMC sample preparation

Dilute the fresh blood samples with PBS buffer and slowly add the diluted blood sample to the upper layer of the Lymphocyte Separation Medium (Beijing Solarbio), centrifuge at 800 g for 20 min, suck the middle layer of PBMC cells and filter them with a membrane filter (100 µM), then wash twice with PBS buffer.

### Antibody

All the antibodies were purchased from Fluidigm, some of them are pre-labeled (with Lanthanide metal) antibodies, and the others are purified antibodies and manually conjugated with metals by using MCP9 or X8 single metal labeling kits (Fluidigm) according to the manufacturer’s instructions. The detailed antibody panel was listed in the Supplementary Table 1.

### Staining of surface proteins and nuclear proteins

Cells in PBMC samples were stained with 0.5 µM cisplatin in PBS without Ca^2+^ and Mg^2+^ at room temperature to distinguish dead from living cells. The cisplatin-stained cells were blocked with FcR Blocking Solution (BioLegend), followed by incubation with a prepared mixture of metal-conjugated antibodies at 4 °C for 30 min. Cells were washed and incubated again for 30 min at 4 °C with the prepared mixture of metal-conjugated nucleoprotein antibodies. After the second round of washing, the cells were fixed with 1.6% formaldehyde solution for 10 min. Then, the cells were incubated with Intercalator-Ir with a final concentration of 125 nM at 4 °C overnight.

### Data acquisition

The stained cells were washed and resuspended with Cell Acquisition Solution (CAS) with the cell concentration at around 1.1 × 10^6^/mL, 10% EQ Beads (EQ beads: CAS = 1:9) were then added to the cell suspension. Adherent cell clumps were filtered using a membrane filter (35 µM), followed by data acquisition on a Helios (Fluidigm) at 300 event/s.

### Data preprocessing

The data were gated to identify cell events and the exclusion of dead cells by using Cytobank (premium.cytobank.cn). The live cells were then gated to separate the CD45^+^ cell for subsequent clustering and high dimensional analyses.

### Dimensionality reduction and clustering

All the FCS files obtained from the last step were exported from cytobank and the data were input as R using the flowCore R language package. Preprocessed data were down-sampled to a maximum of 10,000 cells per sample and merged into a single dataset, followed by arcsine transformation to normalize the data.

For dimensionality reduction and clustering of the data, we performed principal component analysis using variable genes, followed by dimensionality reduction of the data by t-stochastic neighbor embedding (t-SNE) analysis to obtain a visual 2D image. Next, the dataset is clustered by the module optimization algorithm, and each cluster is manually annotated according to the biomarker expression.

### *TP53* gene mutation

Peripheral blood samples sent to Tianjin Hematology Research Institute, then p53 (17p13) probe kit produced by Anbiping Pharmaceutical Technolog (Guangzhou, China) was used to detect *TP53* gene mutation by fluorescence in situ hybridization (FISH).

### Statistical analysis

Significant differences were analyzed using paired Student’s t-test or analysis of variance, and p < 0.05 was considered significant.

## Result

### High-dimensional immunophenotyping of DHL using CyTOF

**Fig. 1:**
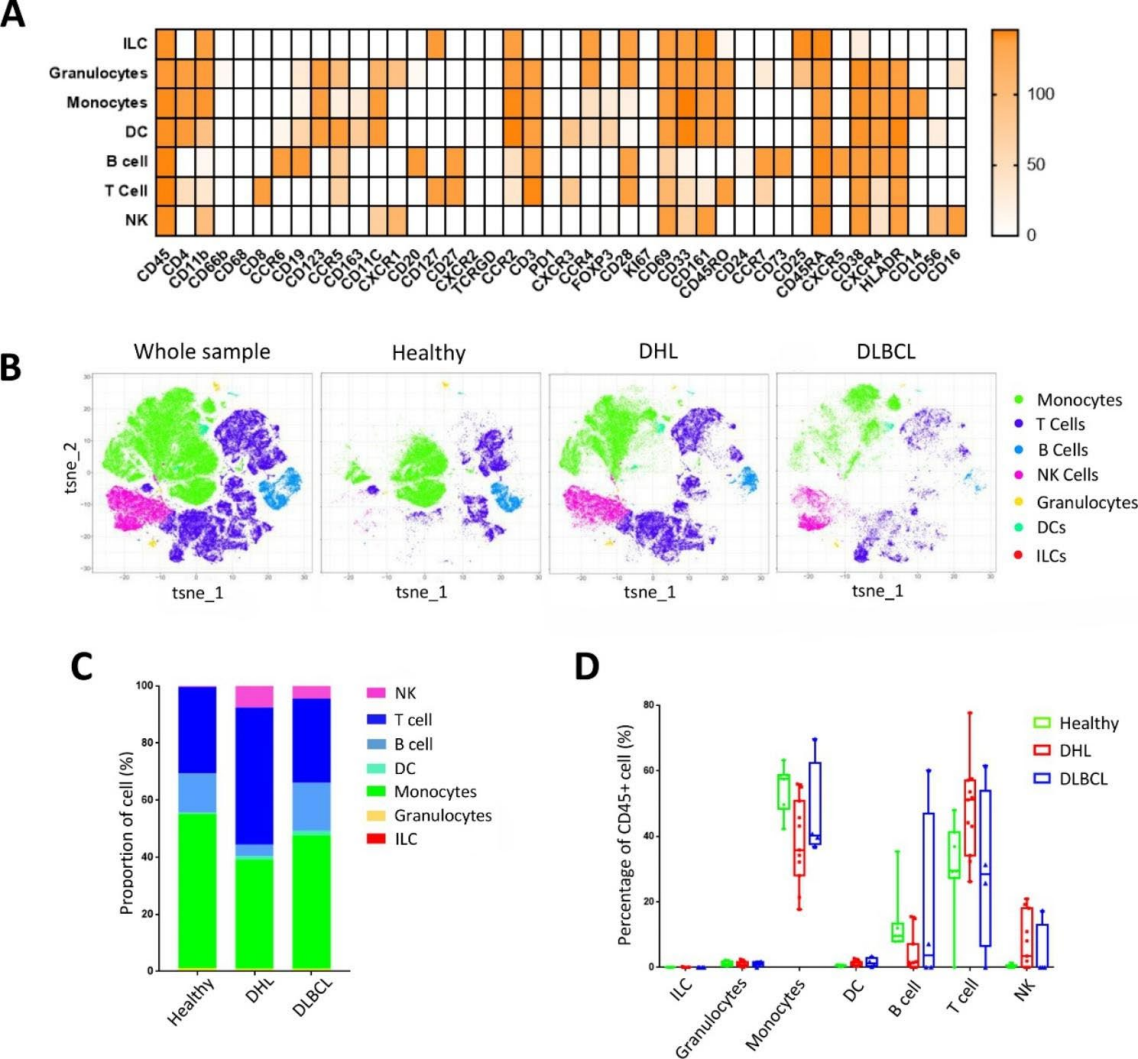
The composition of CD45^+^ immune cells in the three groups. **A**: Heat map of major immune cells in PBMCs depending on the expression of markers; **B**: t-SNE of major immune cells in PBMCs from the whole sample, healthy people, DHL and DLBCL group. Cells are colored basing on cell types. **C**: Relative fractions of major immune cells in PBMC CD45^+^ immune cells from the healthy people, DHL, and DLBCL group. **D**: Percentage of major immune cells in PBMC CD45^+^ immune cells from the healthy people (n = 7), DHL (n = 11), and DLBCL group (n = 4)

The R data package was used to cluster and analyze the CD45^+^ immune cells of all samples in the preprocessed data. According to the expression profile of canonical lineage markers (Supplementary Fig. S1), all immune cell subsets were determined and re-clustered to obtain the marker expression status of seven major immune cell subsets (Fig. 1A). Next, we explored whether the relative frequency of CD45^+^ immune cells accounted for by different cell subsets differed among the three groups of samples,

which was visualized using the t-SNE algorithm to obtain the corresponding projections (Fig. 1B). There are large differences in the composition of immune cells among the three groups of samples (Fig. 1B, C). Compared with the healthy group, the proportions of monocytes (p = 0.0047) and B cells (p = 0.041) were significantly lower in the DHL group, while NKs (p = 0.0044) and T cell (p = 0.03) ratios were notably higher. The ratios of different cell subsets also differed to some extent in the DLBCL group compared with the healthy group, but it’s hard to make a conclusion due to the large heterogeneity in samples. The other two groups showed significant differences (Fig. 1D). Overall, the above results suggest that DHL patients may suffer from more severe immune dysfunction. In addition, the differences among the *MYC/BCL2, MYC/BCL6*, and triple hits patients and the differences of two age groups were further analyzed (Supplementary Fig. S8-9).

### Peripheral circulating T cells in DHL patients

**Fig. 2:**
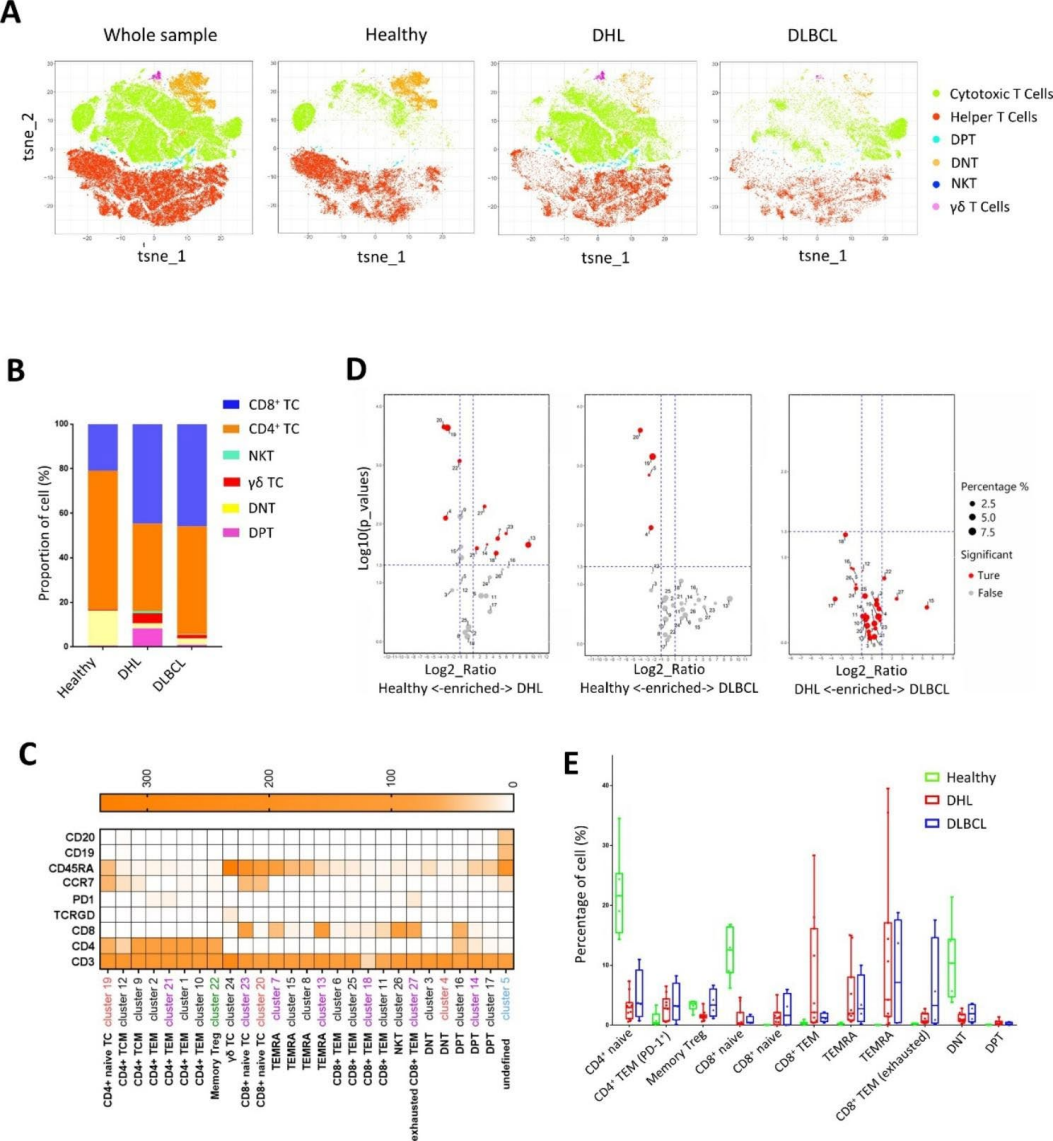
The profile of peripheral circulating T cells in the three groups. **A**: t-SNE projections of major T cells subsets from the whole sample, healthy people, DHL and DLBCL group; **B**: Relative fractions of major subsets in T cells from the healthy people, DHL, and DLBCL group. **C**: Further division of cell subsets in T cells based on the expression of markers. Red, green, blue: the cell subpopulations of the Healthy group whose abundance is significantly higher than that of DHL/DLBCL, DHL or DLBCL, respectively; purple: the cell subpopulations of the DHL group whose abundance is significantly higher than that of the Healthy group; **D**: Volcano plots reflecting the pairwise differences in the abundance of T cell subsets among the three groups of PBMCs, p values of cluster percentage were calculated by unpaired t-test. **E**: Percentage of major T cells subsets in T cells from the healthy people (n = 7), DHL (n = 11), and DLBCL group (n = 4)

According to the expression of surface markers, T cells were classified into six cell subsets. t-SNE was then performed for dimensionality reduction of the whole sample, as well as each group (Fig. 2A), and significant differences in T cell subsets can be seen among the three groups. Among all T cell subsets, the ratio of CD8^+^ cells in the two patient groups were significantly higher than that in the healthy group, while CD4^+^ T cells in the DHL group were comparatively lower. To depict the immune cell profile of peripheral blood in more detail, we also analyzed the ratio of NKT cells, γδT cells, double-negative T cells (DNT), and double-positive T cells (DPT) in each group. NKT cells, γδ cells, and DPT cells accounted for a negligible proportion of T cells in the healthy group, but a higher portion in patient groups. It’s worth noting that the proportion of DPT cells was significantly higher in the DHL group than that in the other two groups, and DNT cells are much rarer in the patient groups than that in the healthy group (Fig. 2B).

According to the expression of CD45RA and CCR7, we further refined the CD4^+^ and CD8^+^ cell subsets into the following subsets: naïve T cells (CD45RA^+^, CCR7^+^), central memory T cells (CD45RA^−^, CCR7^+^), effector memory T cells (TEM; CD45RA^−^, CCR7^−^) and terminally differentiated effector memory T cells (TEMRA; CD45RA^+^, CCR7^−^) [14], whereas CD25^+^ CD127^−^ CD4^+^ T cells were defined as Treg cells (Fig. 2C). After pairwise contrast among the three groups, we found that the healthy group had some cell subsets that displayed significantly higher abundance than either DHL group or DLBCL group (Fig. 2D). There were also some cell subsets in DHL group showed higher abundance than that in the healthy group, however, no cell subsets displayed remarkably different between the DHL and DLBCL group.

After further analysis of the above cell subsets displaying significant differences, we found that both CD4^+^ and CD8^+^ naïve T cells showed significantly higher proportion in the healthy group than that in the patients’ groups. Conversely, CD4^+^ or CD8^+^ TEM cell levels were much higher in the two patient groups (Fig. 2E), and some of these TEM subsets harbored relatively higher expression levels of PD-1 (Fig. 2C). In addition, we also noticed that two CD8^+^ TEMRA cell subsets that were negligible in the healthy group, but much more abundant in the DHL and DLBCL groups, and even showed extremely high levels in some samples (Fig. 2E).

### Peripheral circulating B cells in DHL patients

**Fig. 3:**
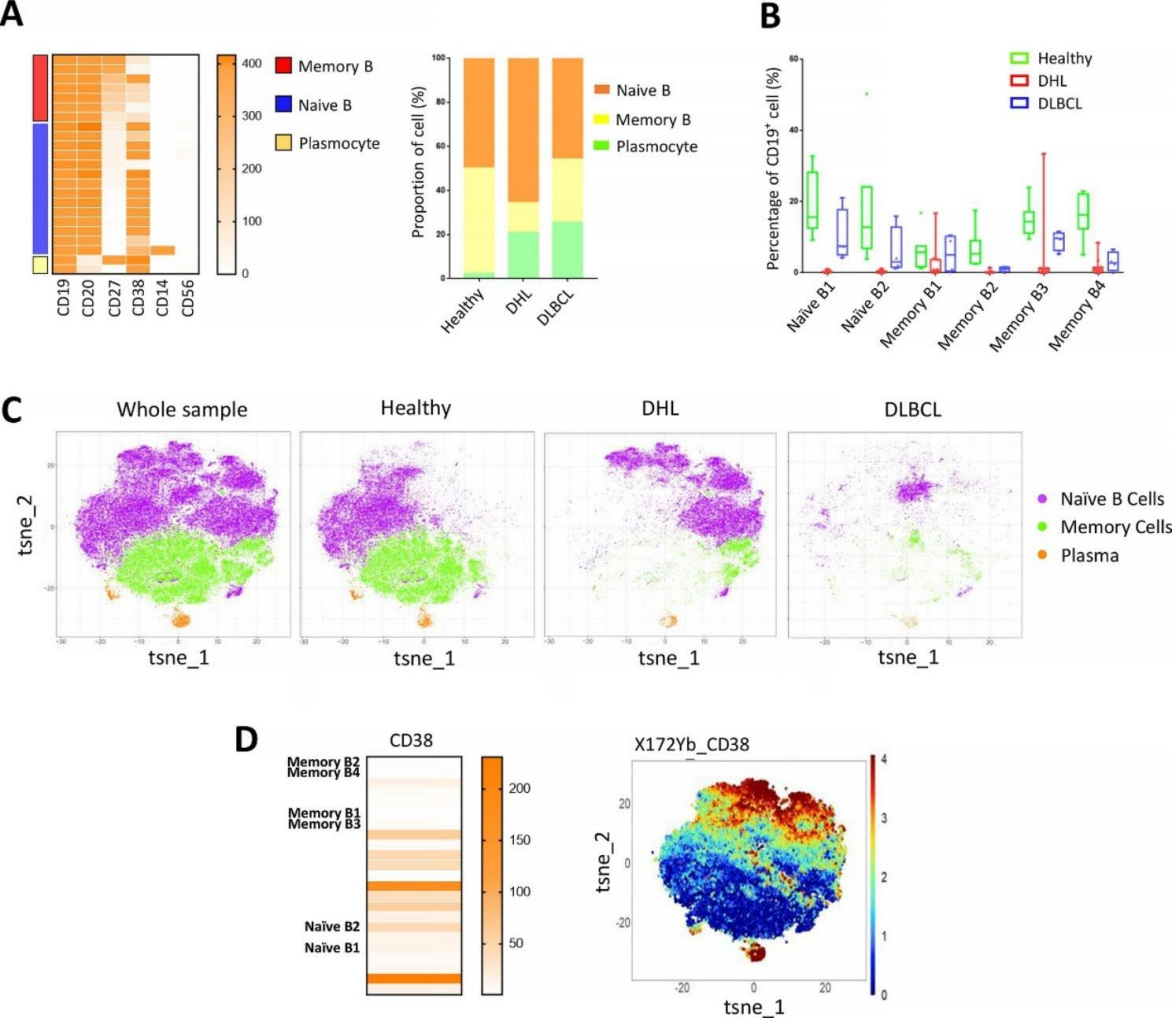
The profile of peripheral circulating B cells in the three groups. **A**: B cells were divided into three major subsets based on the expression of markers, the relative fractions of major subsets in B cells from the healthy people, DHL, and DLBCL group was presented; **B**: Percentage of B cells subsets from the healthy people (n = 7), DHL (n = 11), and DLBCL group (n = 4) that displayed discrepancy between healthy and patient groups; **C**: t-SNE plot of major B cells subsets from the whole sample, healthy people, DHL and DLBCL group. Cells are colored basing on cell types. **D**: CD38 expression heat map of cell subsets in B cells and t-SNE heat map of CD38 in B cells

According to the expression of surface markers, B cells were classified into three subsets: naïve B cells (CD27^−^), memory B cells (CD27^+^) and plasma (CD20^−^, CD38^+^) (Fig. 3A). The analysis showed that the proportion of naïve B cell subsets in the DHL group was significantly higher than that in the healthy group and DLBCL group, while the memory B cell subsets were lower than that in the other two groups (Fig. 3A). Notably, the plasmacyte in the patient groups are much more abundant. All these features indicate a strong humoral immune response in patients. Besides, we found that there were two naïve B cell subsets (naïve B1, naïve B2) and three memory B cell subsets (memory B2, memory B3, memory B4) showed a high discrepancy between the healthy group and DHL group (Fig. 3B). In addition, we found that all these five cell subsets, as well as memory B1 cells, harbored negative or low expression of CD38 (Fig. 3D). However, CD38 was highly expressed in other naïve B cell subsets that are only enriched in DHL group, according to the comparison of the t-SNE cluster map (Fig. 3C) with the CD38 expression heat map (Fig. 3D).

### Disproportion of peripheral circulating monocytes in DHL patients

**Fig. 4:**
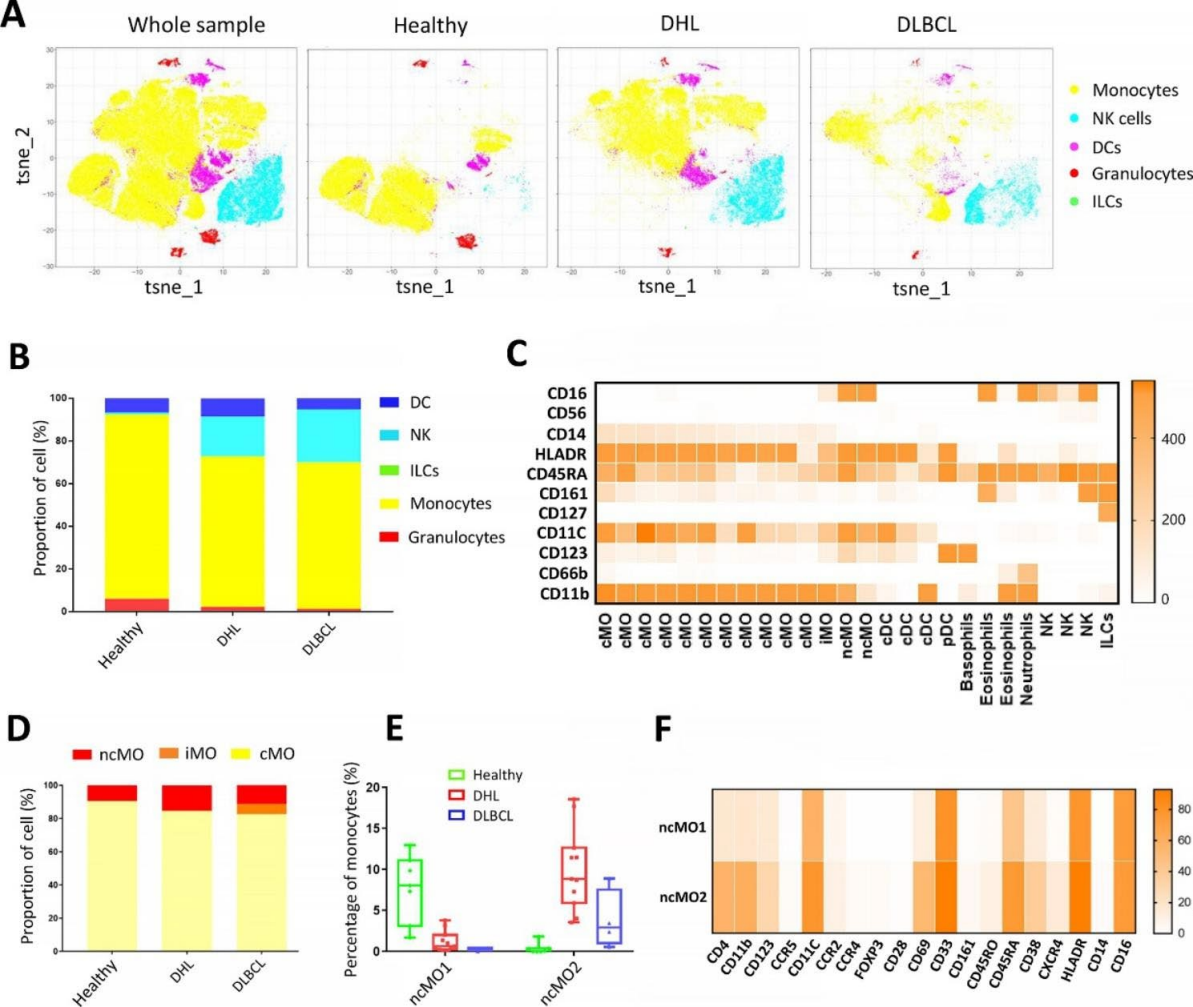
The profile of peripheral circulating monocytes, DCs and NK cells in the three groups. **A**: t-SNE plot of monocytes, NK cells, DCs, granulocytes and ILCs subsets from the whole sample, healthy people, DHL, and DLBCL group. Cells are colored basing on cell types; **B**: Relative fractions of major subsets from the healthy people, DHL, and DLBCL group; **C**: Further division of the major subsets based on the expression of markers; **D**: Relative fractions of major subsets in monocytes from the healthy people, DHL, and DLBCL group. **E**: Percentage of ncMO1 and ncMO2 subsets in monocytes from the healthy people (n = 7), DHL (n = 11), and DLBCL group (n = 4); **F**: Heat map of ncMO1 and ncMO2 subsets exhibiting the expression of major markers

Considering that different cell subsets of monocytes, DCs and NK cells are scattered and have many corresponding markers, we clustered these three groups of cells based on the expression of CD45 regardless of CD3 and CD19, in case of data lose during individual extraction (Fig. 4A, B). The NK cells were negligible in the healthy group,

while accounting for around 20% in the patient groups. Instead, the monocytes in the DHL and DLBCL groups are much less than that in the healthy group.

Furthermore, the cell subsets were classified depending on their markers as shown in Fig. 4C. According to the expression of CD14 and CD16, monocytes can be divided into three subtypes: classical monocytes (cMO, CD14^+^, CD16^−^), intermediate monocytes (iMO, CD14^lo/−^, CD16^+^) and non-classical monocytes (ncMO, CD14^−^, CD16^+^) type (Fig. 4C). As the most common subgroup, cMO was less abundant in the patient group than in the healthy group, while the levels of ncMO and iMO were significantly increased in the patient group (Fig. 4D). However, it was reported that cMO and iMO were enriched in the peripheral blood of DLBCL patients, whereas the abundance of ncMO should be lower [15], which was not consistent with our analysis results. To explore the reason, we further analyzed the two ncMO cell subsets and found that the ratio of the ncMO1 subset was in line with the previous report, whereas another subset, ncMO2, was significantly higher in the DHL and DLBCL groups than that in the healthy group (Fig. 4E), and the large proportion and high enrichment of this subgroup could be the reason that the overall proportion of ncMO is increased in the peripheral blood of DHL and DLBCL patients. Subsequently, we analyzed the signal intensities of the markers used for the profiling of ncMO1 and ncMO2 (excluding markers with no detected signals) with a heatmap, as it was shown, most of the markers were expressed at a higher level on ncMO2 than ncMO1 (Fig. 4F).

### Differences between DCs and NK cells in DHL

**Fig. 5:**
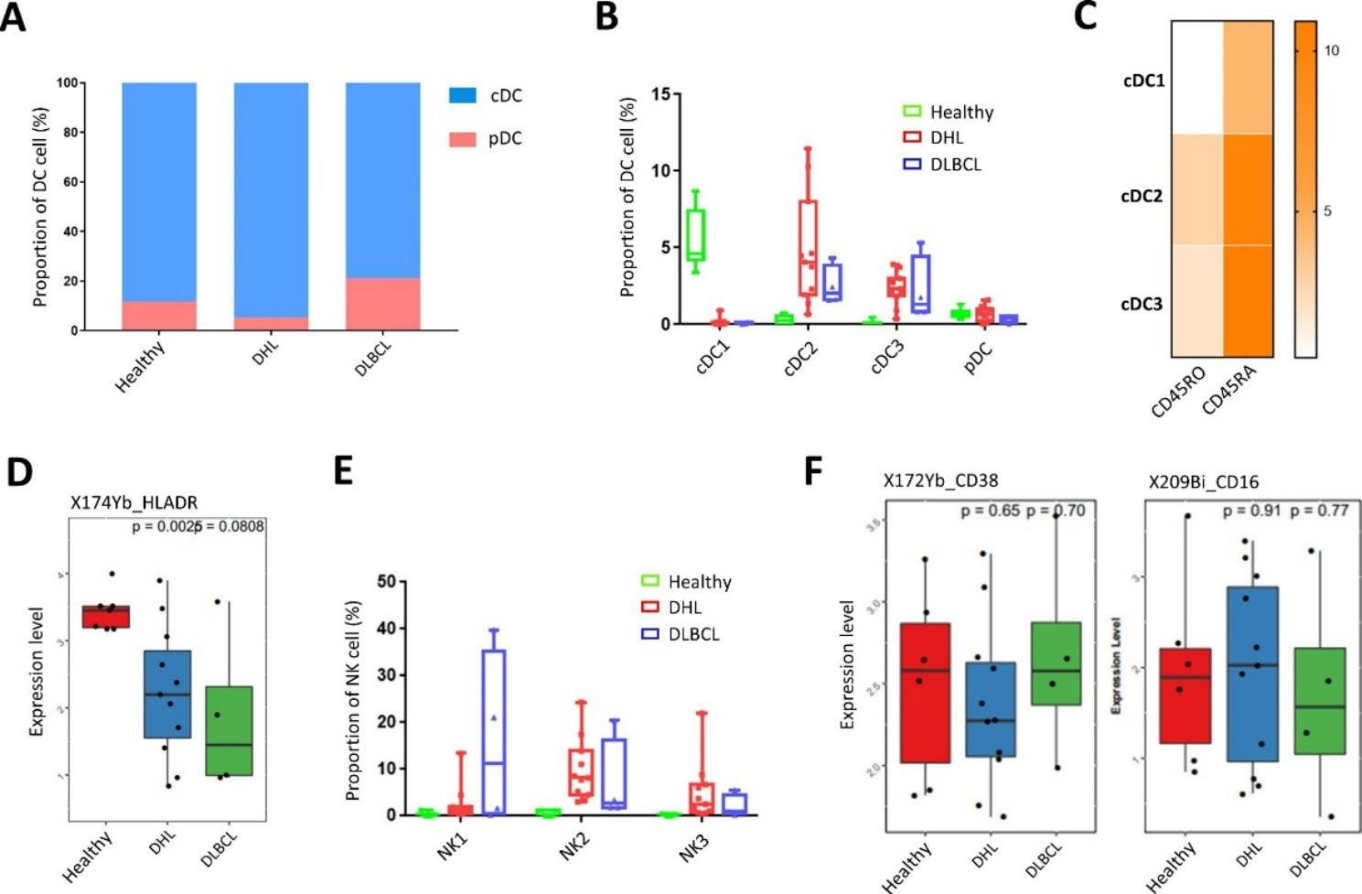
Characterization of DCs and NK cells from mass cytometry data. **A**: Relative fractions of pDCs and cDCs in DCs from the HC, DHL, and DLBCL group; **B**: Percentage of all subsets in DCs from the healthy people (n = 7), DHL (n = 11), and DLBCL group (n = 4); **C**: Heat map of cDCs subsets exhibiting the expression of CD45RA and CD45RO; **D**:Box-plot showing the expression of HLA-DR in the DCs between healthy people, DHL and DLBCL; **E**: Percentage of all subsets in NK cells from the healthy people (n = 7), DHL (n = 11), and DLBCL group (n = 4); **F**: Box-plot showing the expression of CD38 and CD16 in the NK cells between healthy people, DHL and DLBCL.

DC cells are important for the activation of CD4^+^ or CD8^+^ T cells [16, 17]. According to the expression of CD123 and CD11c, DCs were subdivided into two subtypes: conventional DCs (cDCs, CD11c^hi^, CD123^lo^) and plasmacytoid DCs (pDCs, CD11c^lo^, CD123^hi^), the majority was cDCs, no significant difference was observed among the three groups (Fig. 5A). Further analysis found that the three subsets of cDCs (cDC1, cDC2, cDC3) displayed in different abundance between healthy people and patients, cDC1 cells were mainly existed in healthy people, while cDC2 and cDC3 cells were dominantly presented in patents (few differences between DHL and DLBCL groups) and almost absent in healthy people (Fig. 5B). For these cell subsets, we analyzed the CD45RA and CD45RO expression profiles, and found that the expressions of these two markers on cDC2 and cDC3 cells were significantly increased compared to the other subset (Fig. 5C). Besides, we found that the HLA-DR expression in the patient group was decreased compared with the healthy group (healthy vs. DHL, p = 0.0025; healthy.

vs. DLBCL, p = 0.08), indicating that the antigen-presenting ability of the patient DC cells was weakened (Fig. 5D).

In the analysis above, we mentioned that the proportion of NK cells in the patient group was significantly increased. According to the expression of CD56, a biomarker of NK cells, we subdivided NK cells into three subsets: NK1, NK2 and NK3. CD56 and CD16 are the main biomarkers of NK1 (CD56^bright^ CD16^bright^), NK2 (CD56^bright^ CD16^dim^) and NK3 (CD56^dim^ CD16^bright^) cell subsets. NK2 subpopulation is a subset of CD56^bright^ NK cells with low proportion in peripheral blood. it is generally believed that NK2 subpopulation is the precursor of NK3 cell subsets. As for NK3 subpopulation, the dominant NK cell subset in PBMC, has strong cytotoxicity. It is worth noting that NK1 cell subpopulation seems to be a double bright NK subset. However, the definition of NK1 subset needs to be further confirmed due to the lack of other key markers expression. In fact, these NK cell subsets were almost absent in the healthy human peripheral blood samples (Fig. 5E). Surprisingly, CD38 and CD16, two markers of cytotoxic activity, were not significantly different between healthy and patient groups (Fig. 5F) [18, 19].

### The heterogeneity of DHL group

**Fig. 6:**
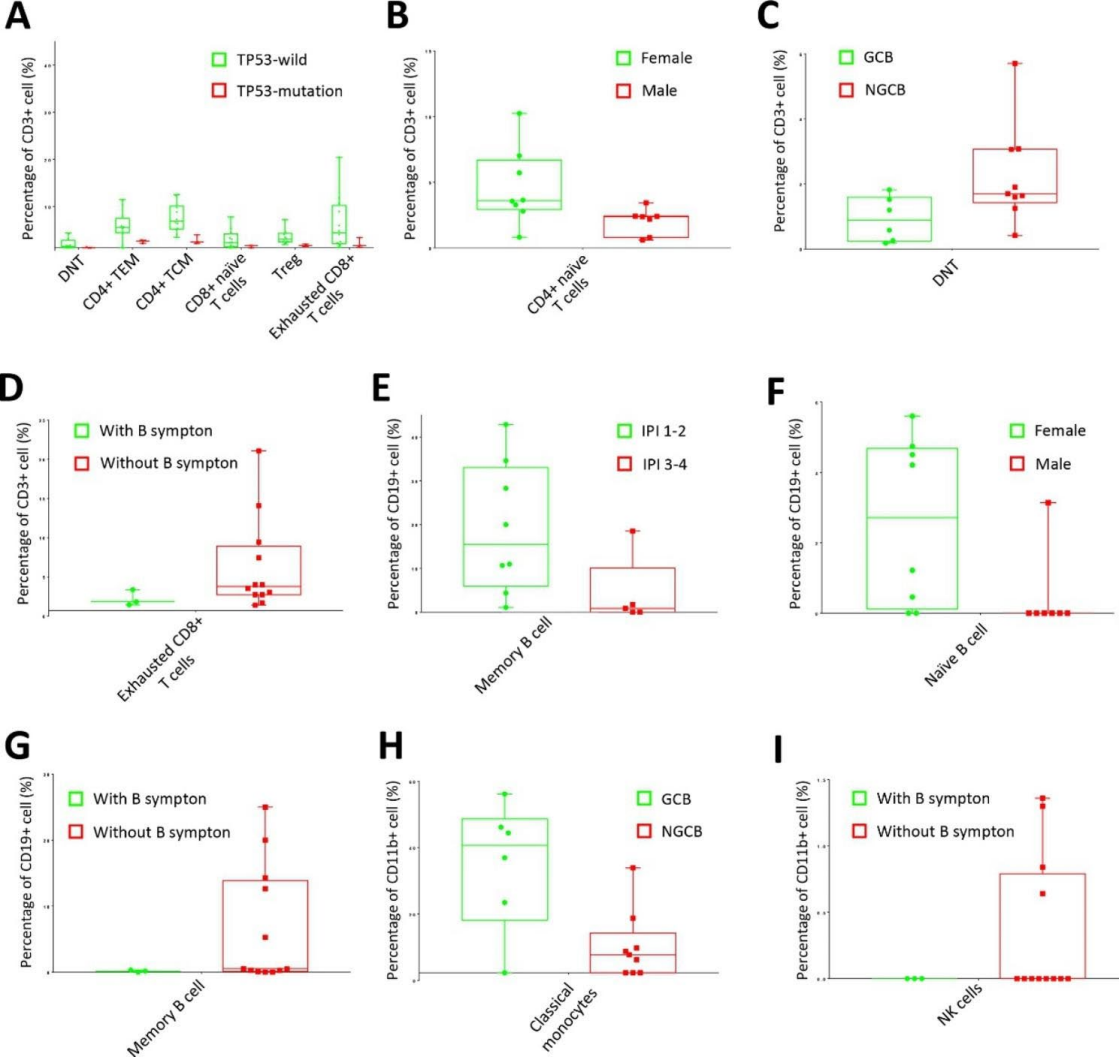
Box-plot displays immune cell subsets with significant differences over clinical characteristics of DHL patients in different immune cell groups

In order to evaluate the heterogeneity in the DHL patient group, we analyze and pairwise compare the immune cell population depended on different clinical characteristics, including sex (female vs. male), COO subtypes (GCB vs. non-GCB), IPI (1 ~ 2 vs. 3 ~ 4), B symptom (with B symptom vs. without B symptom), *TP53* mutation (*TP53* wild *vs. TP53* mutation) and disease stage (I ~ II vs. III ~ IV). Firstly, t-SNE was performed for dimensionality reduction of the whole DHL samples in terms of T cells, B cells and monocytes, to see the profiles of their subtypes, respectively (Supplementary Fig. S2, S4, Fig. S6). Afterwards, t-SNE was further performed to compare the difference of subtypes depending on clinical characteristics under each immune cell group (Supplementary Fig. S2, S4, Fig. S6). The results show that there are some subtypes displayed distinguished enrichment depending on different clinical characteristics. Volcano plotting was then performed to highlight the significantly outlined subtypes in each pairwise comparison (Supplementary Fig. S3, Fig. S5, Fig. S7). After affiliating these subtypes, box plotting was used to compare the enrichment of each subtype in detail (Fig. 6). Compared to the *TP53* mutation group, DNT, Treg, CD4^+^ TEM, CD4^+^ TCM, CD8^+^ naïve T cells and exhausted CD8^+^ T cells displayed higher enrichment in *TP53* wild group (Fig. 6A). CD4^+^ naïve T cells and naïve B cells are significantly more abundant in female patients than in male patients (Fig. 6B, F). Besides, DNT was enriched in non-GCB group, while classical monocytes clearly display higher abundance in GCB group (Fig. 6C, H). Additionally, the DHL patients with B symptom harbored negligible exhausted CD8^+^ T cells, memory B cells and NK cells, and it’s same for most patients without B symptom, however, these subtypes showed remarkably high in certain samples (Fig. 6D, G, I). Furthermore, IPI could be another factor affecting the population of immune cells, for example, memory B cells are significantly more abundant in patients in IPI 1 ~ 2 compared to those in IPI 3 ~ 4 (Fig. 6E).

## Discussion

CyTOF is a powerful high-dimensional single-cell immune system analysis platform that can simultaneously analyze more than 50 markers at the single-cell level. It integrates the advantage of high-speed analysis of traditional flow cytometers and the high-resolution capability of mass spectrometry detection. To date, no study has been done to investigate the comprehensive landscape of peripheral circulating immune cells in DHL patients using CyTOF technology. In this study, we evaluated the PBMC cells extracted from the peripheral blood of DHL patients for high-throughput analysis by CyTOF, and used PBMC samples from healthy people and DLBCL patients as controls. The subsets of peripheral circulating immune cells in patients were determined, and the causes and effects of differences in their abundance were analyzed.

The 5th edition of the World Health Organization Classification of Lymphoid Neoplasms was published in 2022, and only the high-grade B cell lymphoma involving *MYC* and *BCL2* rearrangements was retained in this entity (DHL), while lymphoid neoplasms with dual *MYC* and *BCL6* rearrangements are now classified either as a subtype of DLBCL, NOS or HGBL, NOS according to their cytomorphological features [20]. Nonetheless, all the peripheral blood samples in this study were acquired before 2022, and we still considered the DLBCL with *MYC* and *BCL2* and/or *BCL6* rearrangement as DHL/THL according to the 2016 World Health Organization classification of lymphoma.

In the analysis of T cells, we found the absolute number of TEM cells and TEMRA cells were elevated in patients, and increased expression of PD-1 was observed in several TEM subsets. CD8^+^ TEMRA cells are associated with immunosenescence and have been found at a high level in age-related chronic inflammatory diseases [14], whilst PD-1 is often regarded as a biomarker of T cell exhaustion, all of which suggests that T cell population of the patients was ongoing with cellular senescence and depletion. We also noticed that the portion of double-negative T cells (DNT) significantly declined in patients. DNTs is a kind of natural suppressor (NS) cells which was characterized as abnormal regulatory T cells with αβ-TCR and CD25 expression but no CD4 or CD8 expression [21, 22]. Despite the small portion of DNTs in peripheral lymphocytes, it had been demonstrated that DNTs have strong suppressive ability towards CD8^+^ T cells, CD4^+^ T cells, B cells and NK cells in vitro and in vivo, which leads to xenograft or allograft transplantation tolerance and powerful prevention of GVHD (graft-versus-host disease) [23, 24]. DNTs was also proved to play a role of tumor suppressor with the ability to suppress the tumor growth of lung cancer and inhibit cell proliferation of acute myeloid leukemia [25–27]. In addition, Fang et al. demonstrated that the growth of non-small cell lung cancer could be inhibited in vitro by using a combination of PD-1 blockade and DNT cell therapy [28]. Considering T cell depletion occurred in both DHL and DLBCL patients, as well as the tumor suppressor role of DNT cells, the level of DNT cells could be an indication of the prognosis of DHL, and DNT cells and their derivatives can be novel therapeutic agents as monotherapy or combination with PD-1 blockade for the treatment of DHL. Furthermore, significant enrichment of DPT cell subsets and Treg cell subsets were detected in the DHL sample compared to the DLBCL group and healthy group, while DPT had been found in various cancers, including T-cell lymphomas, with abnormally high abundance [29–31].

Among the B cell subsets, the proportion of plasma cell subsets in patients was significantly higher than that in healthy people, indicating that a severe humoral immune response occurred in patients. We also detected a cohort of B cells with negative expression of CD38 that was significantly different in enrichment between DHL patients and the other two groups. Of particular note, CD38 is highly expressed in two naïve B cell subsets that are only enriched in DHL patients. As a type II transmembrane glycoprotein, the highly expressed CD38 is an important factor in the poor prognosis of B cell lymphoma [32, 33]. This suggests that CD38 could be a biomarker for diagnosis (distinguishing from other DLBCL subtypes) or prognosis of DHL. The cancerous B cells of DHL may originally be derived from these naïve B cells and display high expression of CD38, therefore, CD38 could be a potential therapeutic target of DHL [34], but need further validation by proper models, such as PDX model.

As regards to monocytes, research has shown that classic monocytes are important for the initial inflammatory response, while non-classical monocytes are widely believed to have anti-inflammatory effects [35, 36]. In this analysis, we found a non-classical monocyte subset inconsistent with existing reports [15], namely the ncMO2 cell subset, which was abnormally and significantly elevated in patient samples. For this subpopulation, we delved into the expression of its surface antigens, and most of the antibody markers we used (including CD45RA and CD45RO) showed higher levels than that in the other subpopulation (ncMO1). Coincidentally, we also found that cDC2 and cDC3 were remarkably abundant in patients but negligible in the healthy group, and were also accompanied by higher expression of CD45RA and CD45RO. The origin and functional mechanism of ncMO2, cDC2 and cDC3, which are DHL-related monocytes subsets, need to be further studied. Furthermore, the NK cell ratio was significantly elevated in DHL and DLBCL groups, which is different from the previous report that NK cells are subjected to exhaustion and at the low level in newly diagnosed DLBCL patients [37], further investigation should be done in the next stage with dynamic blood samples.

DHL is generally considered as highly heterogeneous cancer in terms of genetic alteration and protein expression profile, and it is still true when it comes to immune cells landscape. We demonstrated the heterogeneity of DHL by analyzing the discrepancy of immune cell population within the DHL cohorts based on the clinical characteristics. Among them, *TP53* is a critical factor contributing to the heterogeneity. Loss of p53 function in lymphoma could results in profound alteration in the transcription of some vital genes and secretion of chemokine/cytokine, leading to great influence on the immune cells population and function [38]. Therefore, it is reasonable that the DHL patients harboring *TP53* mutation associated with reduced population of CD4^+^ TEM and CD4^+^ TCM, since p53 plays a key role in regulating the polarization and differentiation of CD4^+^ T cells [39]. The reduced exhausted CD8^+^ T cell in the *TP53* mutation cohort may due to the declined expression of PD-1, whereas PD-1 is a key co-inhibitory receptor in the process of T cell exhaustion and the activation of PD-1 is mediated by p53 [40, 41]. Surprisingly, the Treg cells level was decreased in *TP53* mutation cohort, while *TP53* loss were usually associated with expansion and filtration of Treg in cancers, and facilitated the tumor progression [42, 43]. In addition, the memory B cells were decreased in the DHL cohort with IPI 3 ~ 4, indicating the deficiency of immune function in these high-risk patients.

Although comprehensive and deep analysis were performed with PBMC sample by CyTOF, and some valuable clues had been unearthed, whether these information from PMBC sample truly reflect the profile of the tumor microenvironment of DHL still need to be further confirmed with tissue specimens. In addition, extending the horizontal and longitudinal research scales with large sample size (both of DHL and r/r DLBCL cohort) and dynamic samples could provide more accurate understanding of DHL.

## Conclusion

We analyzed the peripheral blood samples from eleven DHL patients by CyTOF to characterize the immune profile and expect to discover biomarkers for pharmaceutical and clinical application. Despite the small size of samples, the data of this study are promising as statistically significant differences were observed among DHL cohorts, DLBCL cohorts and the healthy control. The results obtained suggested that DHL patients suffered immune dysfunction according to the changing of immune cell population, and some of the biomarkers, such as CD38, could be useful in diagnosis of DHL, or even be potential therapeutic agents or targets. Nevertheless, further studies with larger sample size, dynamic samples or in vivo models are required to confirm these results. In addition, we also demonstrated the heterogeneity of immune cells in DHL patients, and the status of *TP53* is one of the biggest influencing factors. Overall, this study could provide primary guidance for the diagnosis and treatment of DHL patients.

## Electronic supplementary material

Below is the link to the electronic supplementary material.

Supplementary Material 1

## Data Availability

All data generated or analysed during this study are included in this published article and its supplementary information files.

## List of abbreviations

DHL: Double-hit lymphoma
DLBCL: Diffused large B-cell lymphoma
CyTOF: Cytometry by time of flight
TME: Tumor microenvironment
FISH: Fluorescence in situ hybridization
PBMC: Peripheral blood mononuclear cells
t-SNE: t-stochastic neighbor embedding
DNT: double negative T cell
DPT: double positive T cell
TEM: effector memory T cells
TEMRA: terminally differentiated effector memory T cells
TCM: central memory T cell
cMO: classical monocytes
iMO: intermediate monocytes
ncMO: non-classical monocytes
HGBL: high grade B-cell lymphoma
