# Supplementary material for: Peripheral immune cell profiling of double-hit lymphoma by mass cytometry
Source: BMC Cancer. 2023 Feb 23;23:184. doi: 10.1186/s12885-023-10657-0 (PMC9948356; doi:10.1186/s12885-023-10657-0)
Supplement: Supplementary file 1 — Supplementary Material 1 [file 12885_2023_10657_MOESM1_ESM.pdf]

Supplementary Material

Supplementary Table 1. Antibodies panel in this study

Target	Clone	Source	Label
CCR2	K036C2	Fluidigm	153Eu
CXCR4	12G5	Fluidigm	173Yb
CCR6	G034E3	Fluidigm	141Pr
CXCR3	G025H7	Fluidigm	156Gd
CCR4	L291H4	Fluidigm	158Gd
CCR5	NP6G4	Fluidigm	144Nd
CD33	WM53	Fluidigm	163Dy
CD123	6H6	Fluidigm	143Nd
HLA-DR	L243	Fluidigm	174Yb
CD11C	\	Fluidigm	146Nd
CD127	A019D5	Fluidigm	149Sm
CXCR5	RF8B2	Fluidigm	171Yb
FOXP3	259D/C7	Fluidigm	159Tb
CD69	FN50	Fluidigm	162Dy
Ki67	B56	Fluidigm	161Dy
PD1	EH12.2H7	Fluidigm	155Gd
CD28	CD28.2	Fluidigm	160Gd
CCR7	G043H7	Fluidigm	167Er
CD45RA	HI100	Fluidigm	170Er
CD45RO	UCHL1	Fluidigm	165Ho
TCRgd	11F2	Fluidigm	152Sm
CD3	UCHT1	Fluidigm	154Sm

CD161	HP3G10	Fluidigm	164Dy
CD14	M5E2	Fluidigm	175Lu
CD163	GHI/61	Fluidigm	145Nd
CD16	3G8	Fluidigm	209Bi
CD56	HCD56	Fluidigm	176Yb
CD27	LG.3A10	Fluidigm	150Nd
CD38	HIT2	Fluidigm	172Yb
CD24	ML5	Fluidigm	166Er
CD73	AD2	Fluidigm	168Er
CD25	2A3	Fluidigm	169Tm
CD19	HIB19	Fluidigm	142Nd
CD45	HI30	Fluidigm	89Y
CD20	2H7	biolegend	148Nd
CD68	Y1/82A	biolegend	114Cd
CD66b	G10F5	biolegend	113Cd
CD4	RPA-T4	biolegend	111Cd
CD8	RPA-T8	biolegend	116Cd
CD11B	ICRF44	biolegend	112Cd
CXCR2	48311	R&D	151Eu
CXCR1	42750	R&D	147Sm

Supplementary Table 2. The therapies used for DHL patients and range from previous CR to the relapse time.

Patients	Immuno-chemotherapy [1]	Date of the previous CR	Date of sample acquisition [2]	PFS (month) after CR
DHL001	(R-CHOP ×1) + (R-EPOCH ×3) + (R2-CHOP ×1)	2017.01.20	2020.12.02	46.5
DHL002	(R-CHOP ×2) + (R2-CHOP×2) + (R-CHOP ×1)	2019.08.30	2020.12.31	16
DHL003	(R-CHOP ×1) + (R-EPOCH ×6)	2020.04.29	2021.01.09	9
DHL004	(R-CHOP×1) + (R-EPOCH×4)	2019.04.18	2021.01.20	21
DHL005	(R-EPOCH ×6) + (R×2)	2019.01.04	2021.01.26	24.5
DHL006	(R-CHOP×8) + (R×2)	2020.01.10	2021.02.03	12.5
DHL007	(R-CHOP ×8) + (R×2)	2018.04.15	2021.02.21	34
DHL008	(R-CHOP ×1) + (R-EPOCH×5)	2019.07.18	2021.03.25	20
DHL009	R-CHOP×6	2016.08.23	2021.04.02	55
DHL010	(R-CHOP×2) + (R2-CHOP×4)	2018.01.16	2021.05.26	40
DHL011	(R-CHOP ×8) + (R×2)	2020.04.27	2021.07.03	14

[1]The whole immunochemotherapy used to achieve CR and the cycles of each therapy. R: Rituximab; R-CHOP: Rituximab, Cyclophosphamide, Doxorubicin hydrochloride, Vincristine, Prednisone; R-EPOCH: Rituximab, Etoposide, Prednisone, Vincristine, Cyclophosphamide, Doxorubicin

hydrochloride; R2-CHOP: Lenalidomide plus R-CHOP. 2The date of sample acquisition was considered as the relapse time which was used for calculating PFS.

FigureS1

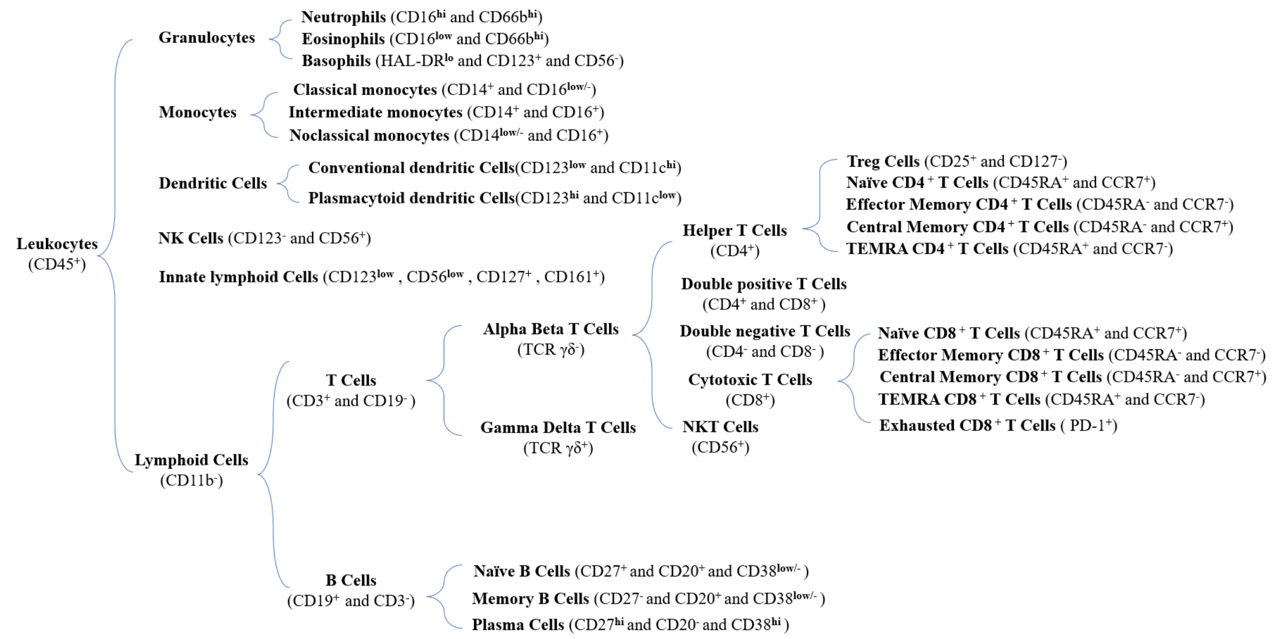

Supplementary Figure S1. The classification of immune cells depending on the expression of markers.

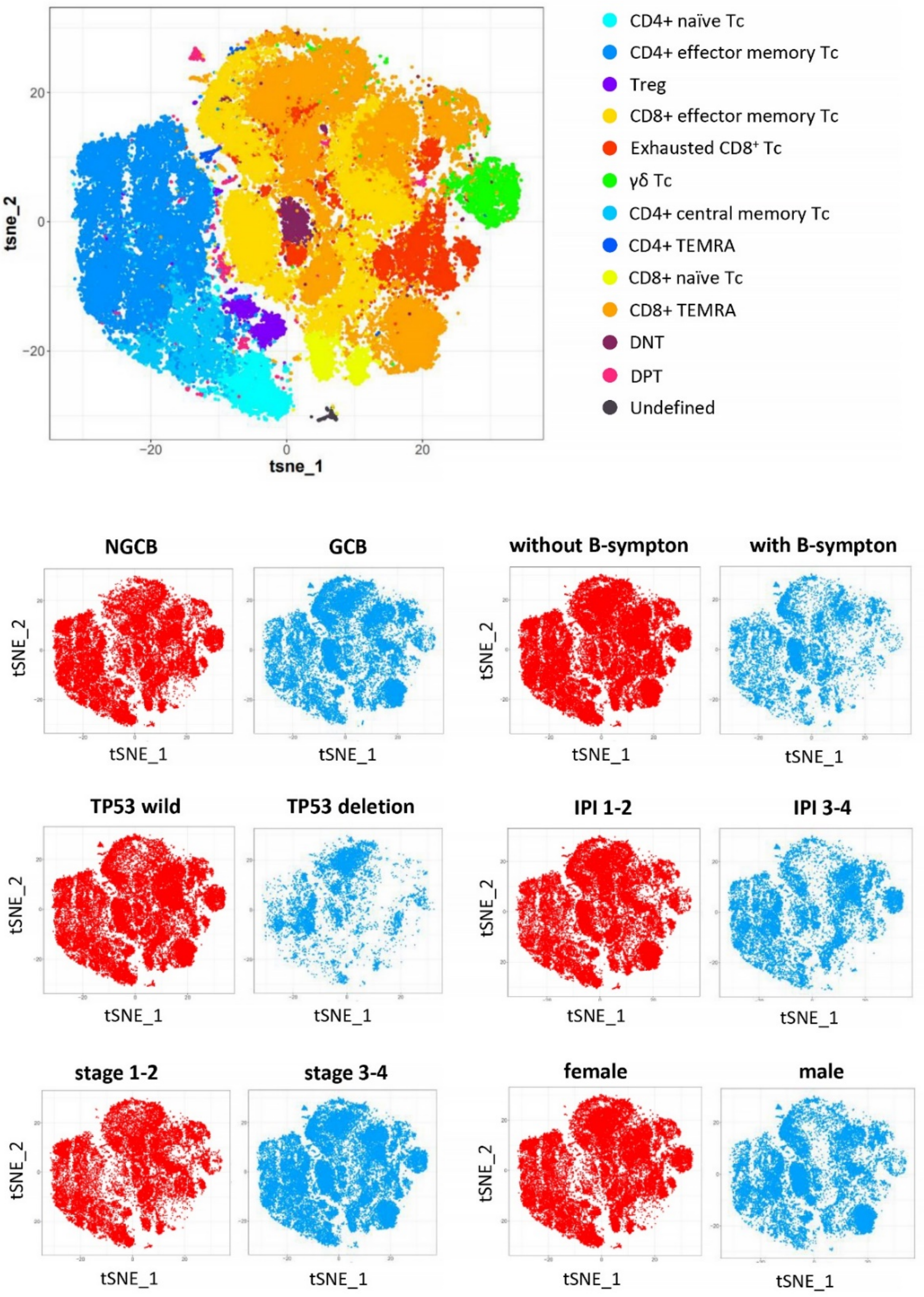

Supplementary Figure S2. T-distributed stochastic neighbor embedding (t-SNE) plot of $CD3^+$ cell subsets (T cells) of the whole DHL samples, and differences of cells subtypes distribution over clinical characteristics. Cells are colored based on cell types or groups.

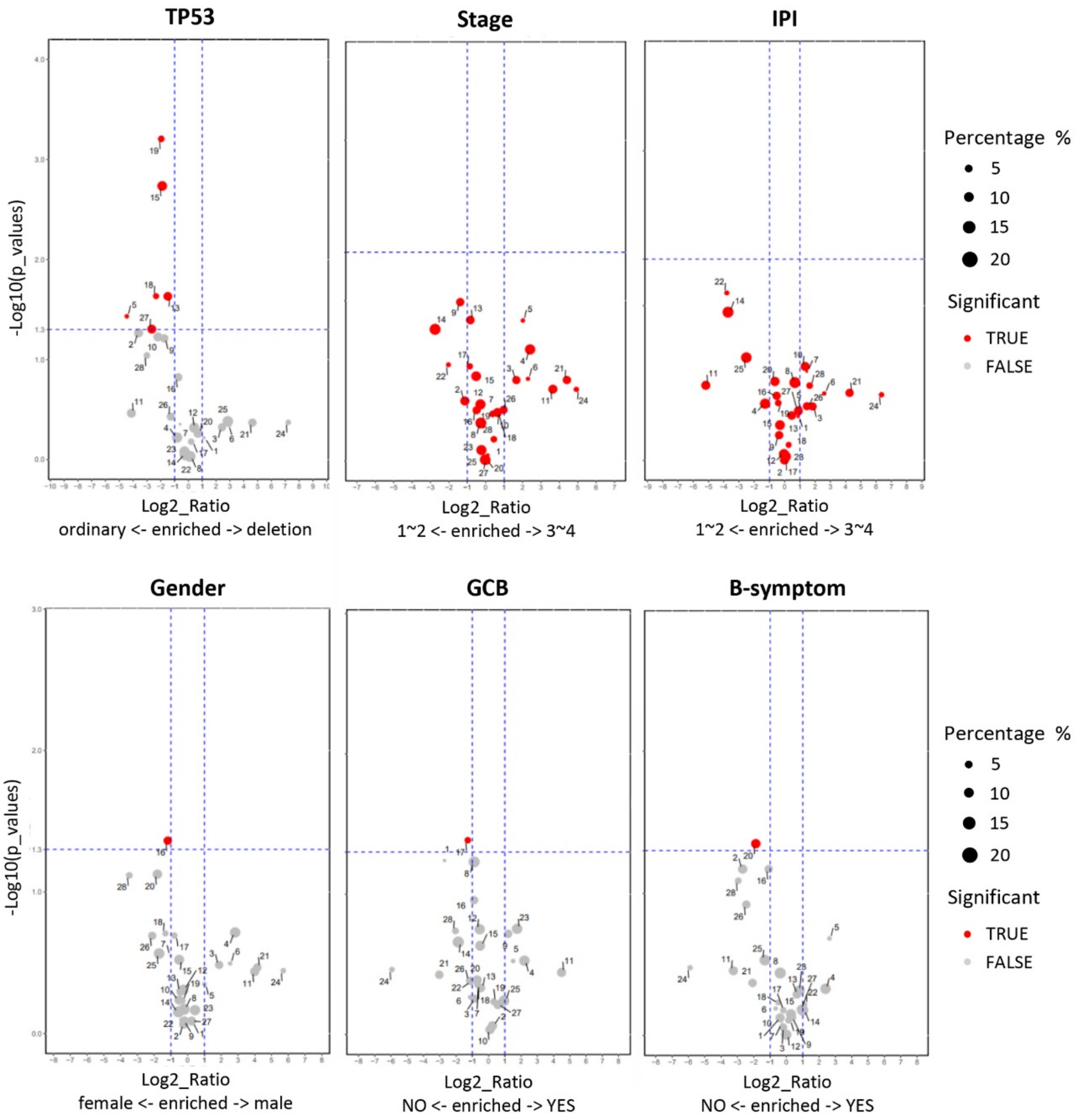

Supplementary Figure S3. Volcano plots reflecting the pairwise differences in the abundance of $CD3^+$ cell subsets over the six clinical characteristics, p values of cluster percentage were calculated by unpaired t-test. TP53 group: cluster5 (defined as DPT), cluster13 (defined as $CD4^+$ effector memory Tc), cluster15 (defined as $CD4^+$ central memory T cell), cluster18 (defined as $CD8^+$ naïve T cell), cluster19 (defined as Treg) and cluster27 (defined as exhausted $CD8^+$ T cell) show significant differences in normal or TP53 deletion patients; Gender group: cluster16 (defined as $CD4^+$ naïve T cell); GCB/NGCB: cluster17 (defined as DNT); B symptom: cluster20 (defined as exhausted $CD8^+$ T cell).

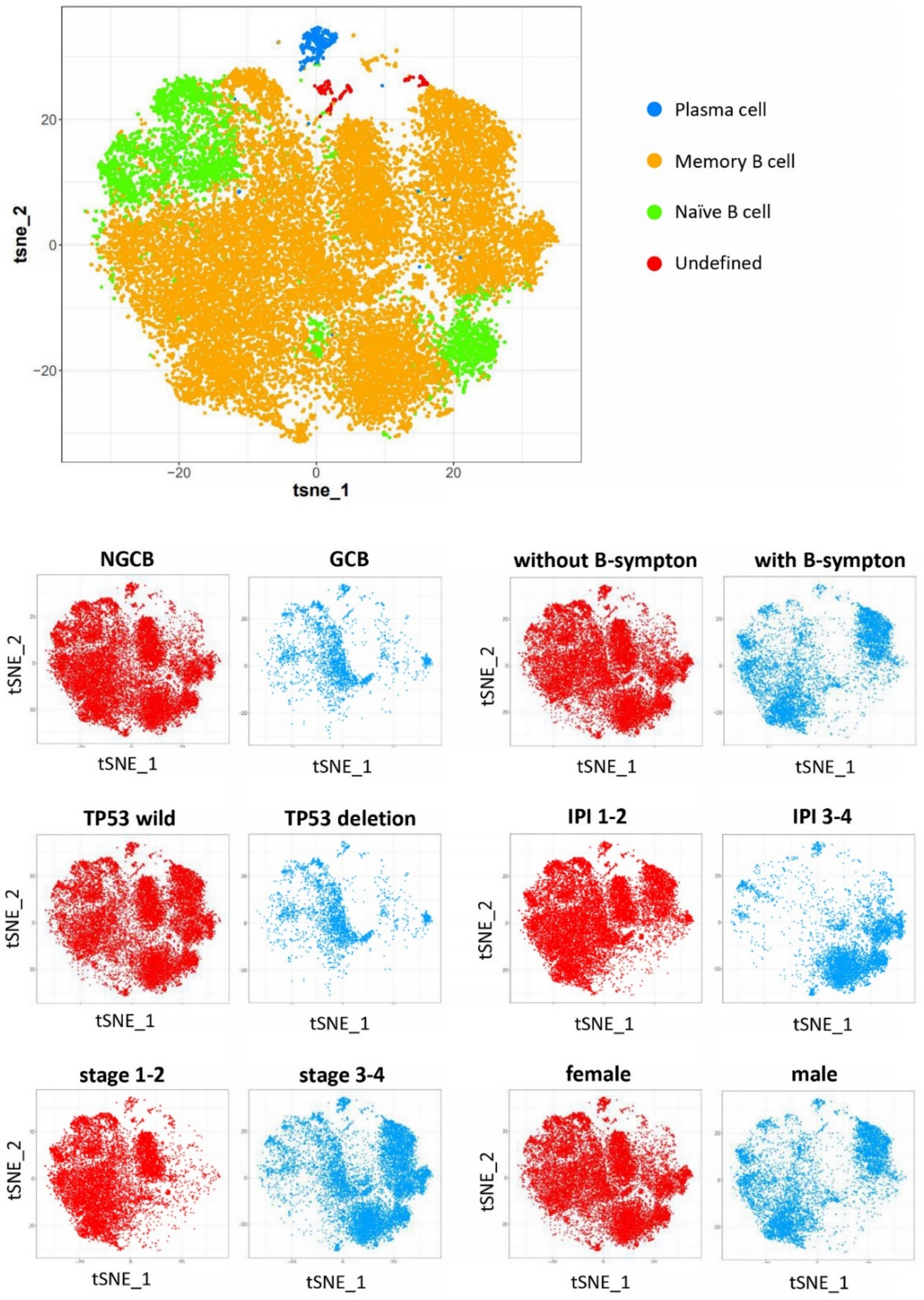

Supplementary Figure S4. T-distributed stochastic neighbor embedding (t-SNE) plot of CD19^{+} cell subsets (B cells) of the whole DHL samples, and differences of cells subtypes distribution over clinical characteristics. Cells are colored based on cell types or groups.

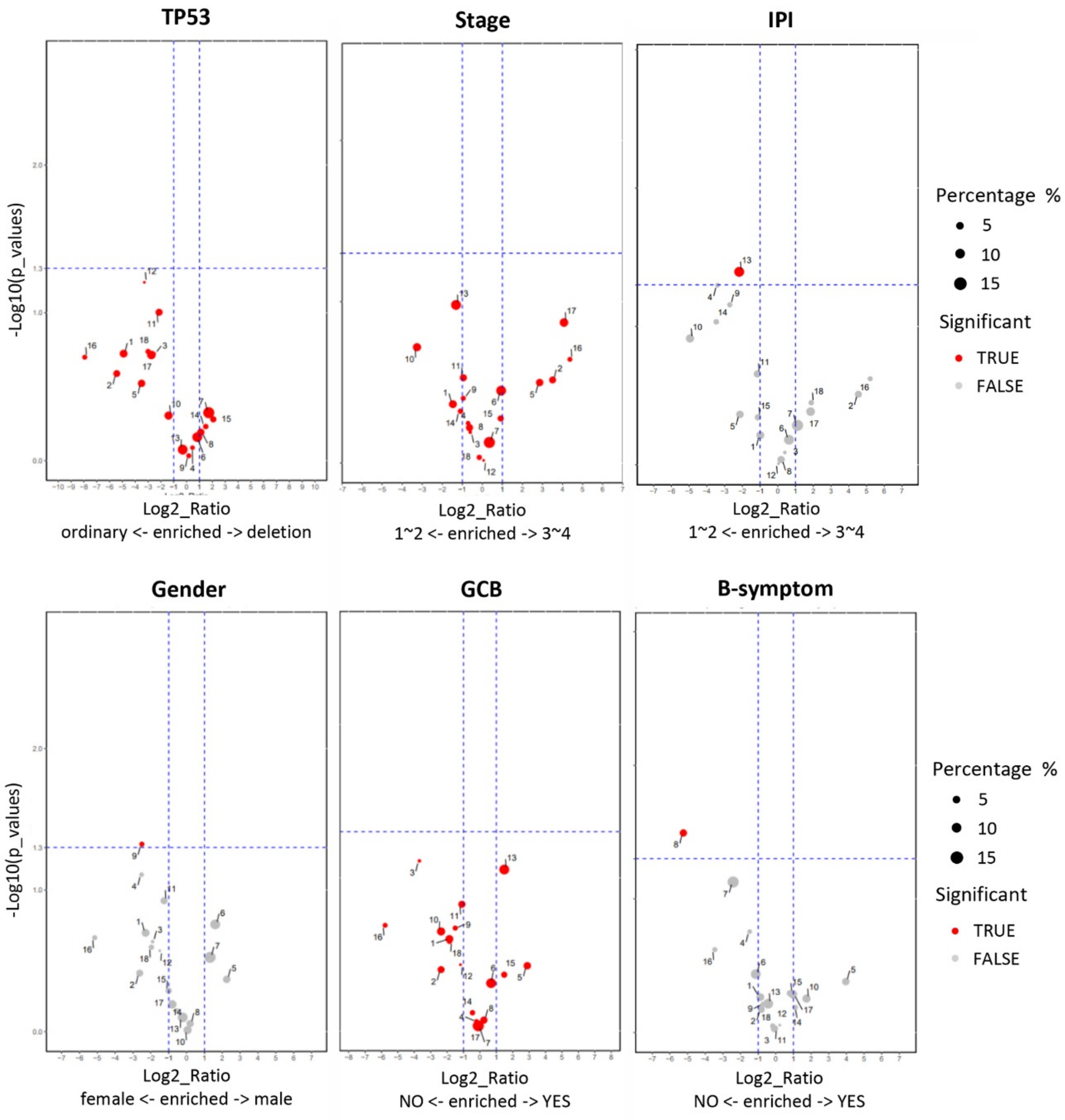

Supplementary Figure S5. Volcano plots reflecting the pairwise differences in the abundance of $CD19^+$ cell subsets over the six clinical characteristics, p values of cluster percentage were calculated by unpaired t-test. IPI group: cluster13 (defined as memory B cell); Sex group: cluster9 (defined as naïve B cell); B-symptom: cluster8 (defined as memory B cell).

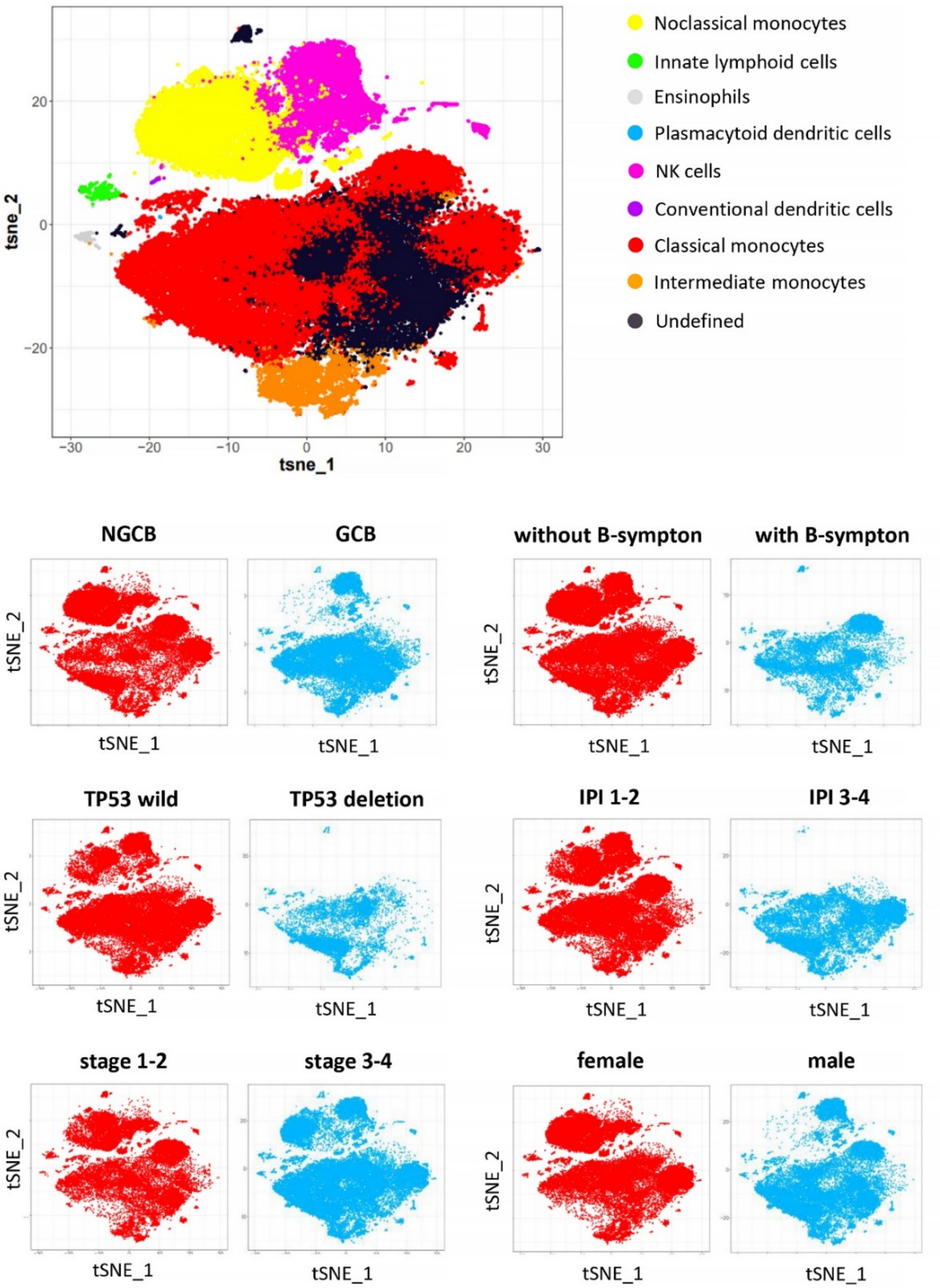

Supplementary Figure S6. T-distributed stochastic neighbor embedding (t-SNE) plot of $CD11b^+$ cell subsets (monocytes) of the whole DHL samples, and differences of cells subtypes distribution over clinical characteristics. Cells are colored based on cell types or groups.

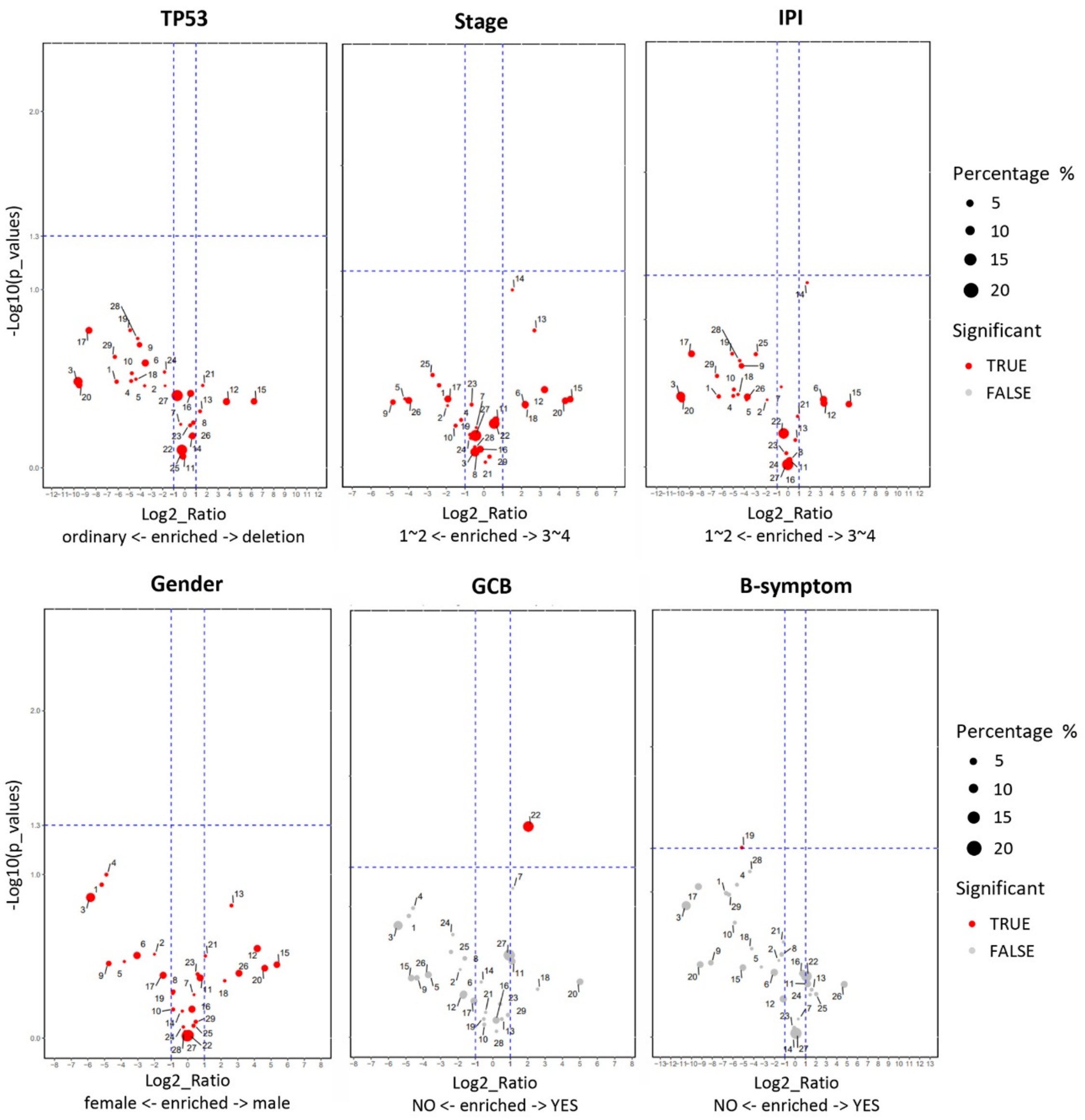

Supplementary Figure S7. Volcano plots reflecting the pairwise differences in the abundance of CD11b^{+} cell subsets over the six clinical characteristics, p values of cluster percentage were calculated by unpaired t-test. GCB/NGCB: cluster22 (defined as classical monocytes); B-symptom: cluster19 (defined as NK cell).

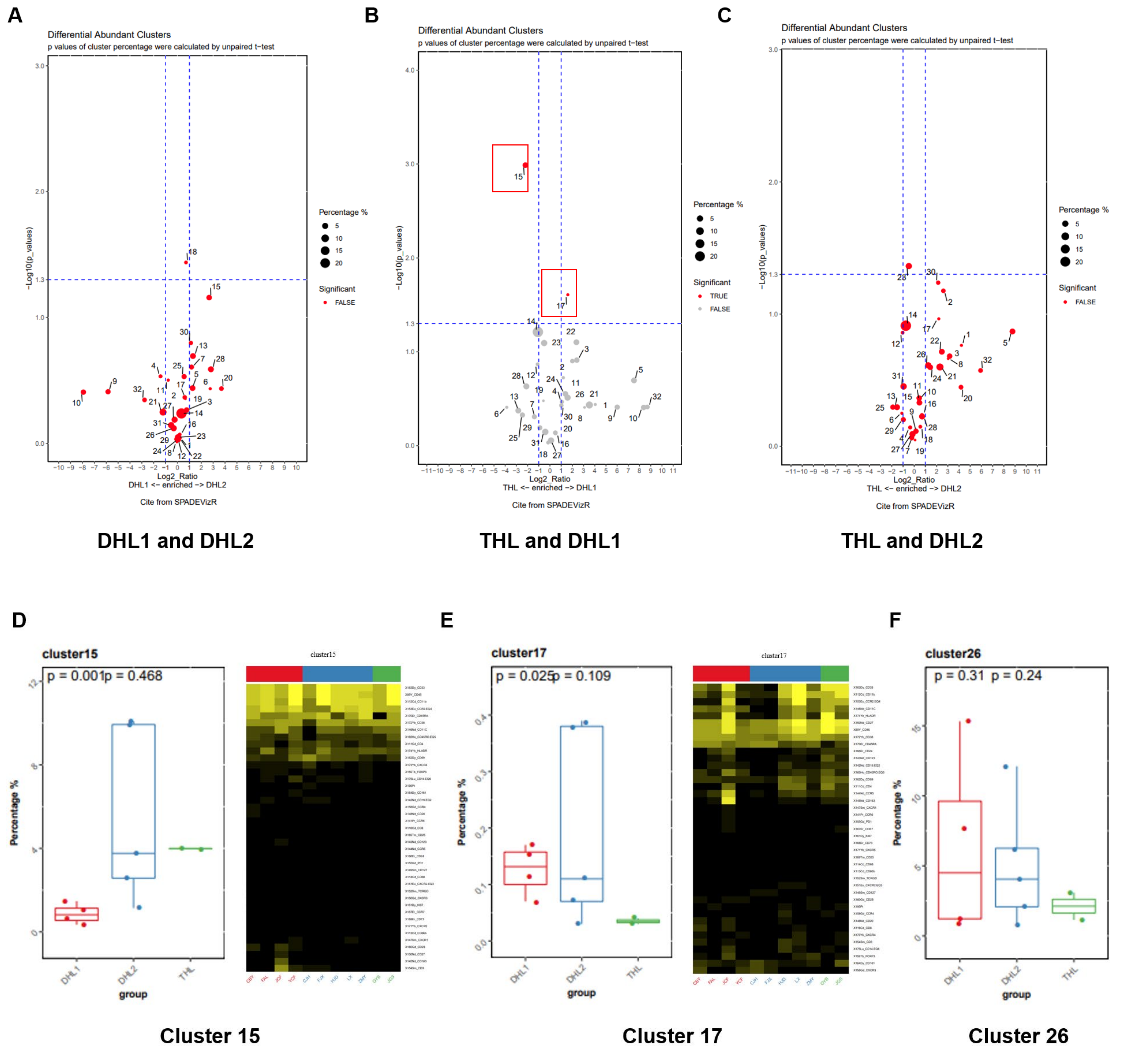

Supplementary Figure S8. Difference analysis of cell subsets in MYC/BCL2, MYC/BCL6 and triple hit patients. **(A, B and C)** Cell subpopulations with significant differences between the three groups of patients are shown in volcano plots. DHL1: MYC/BCL2 translocation DHL patients; DHL2: MYC/BCL6 translocation DHL; THL: triple hit lymphoma patients. **(D, E and F)** Differences in the proportions of cluster15 (DC), cluster17 (NK CD56^{bright}) and cluster 26 among the three groups of patients. The heat maps show the expression of all detected proteins in cluster15 and cluster17 in each patient, DHL1 is marked in red, DHL2 is marked in blue, and THL is marked in green.

The differences among the MYC/BCL2, MYC/BCL6, and triple hits patients were further analyzed. Patients were divided into three groups for analysis and comparison, DHL1 (MYC/BCL2), DHL2 (MYC/BCL6), and THL (triple hits) group, respectively. Our results show that the abundance of cluster 15 (DC cell subpopulation) and 17 (NK bright cell subpopulation) was found to be significantly different between THL and DHL1 patients (**Figure S** A). BCL6 is one of the key factors

regulating the maturation of DC cells (Exp Cell Res. **2006**, 312(8):1312-22; PLOS One. **2014**, 9(6): e101208). Therefore, BCL6 translocation in THL patients may be responsible for the significantly increased abundance of DC subsets (Figure S8).

Furthermore, NK dim subpopulation is further analyzed. Studies have shown that the NK dim subpopulation ($CD56^{dim}$, $CD16^{bright}$) is high in peripheral blood and exhibits classic cytotoxicity (Biosci Rep. **2021** Jul 30;41(7): BSR20210600). The precursor of the NK dim subgroup, NK bright ($CD56^{bright}$, $CD16^{-}$), accounts for only 5%-10% of peripheral blood (J Immunol. **2016** Apr 1;196(7):2923-31). Increased numbers of NK bright cell subsets are reported in most multiple sclerosisdisease treatments (J Clin Med. **2020** May; 9(5): 1450). Our analysis results suggest that the lower abundance of NK bright subpopulation may be one of the reasons why THL has a higher degree of malignancy than DHL (J Clin Pathol, **2020** Mar;73(3):126-138; Mod Pathol, **2018**, 31(9):1470-1478). However, due to the high heterogeneity of the samples, the NK dim subcluster (cluster 26), which plays a dominant role in cell killing, was not significantly reduced in abundance in THL patients (Figure S8). Therefore, further expansion of the sample size for analysis is necessary.

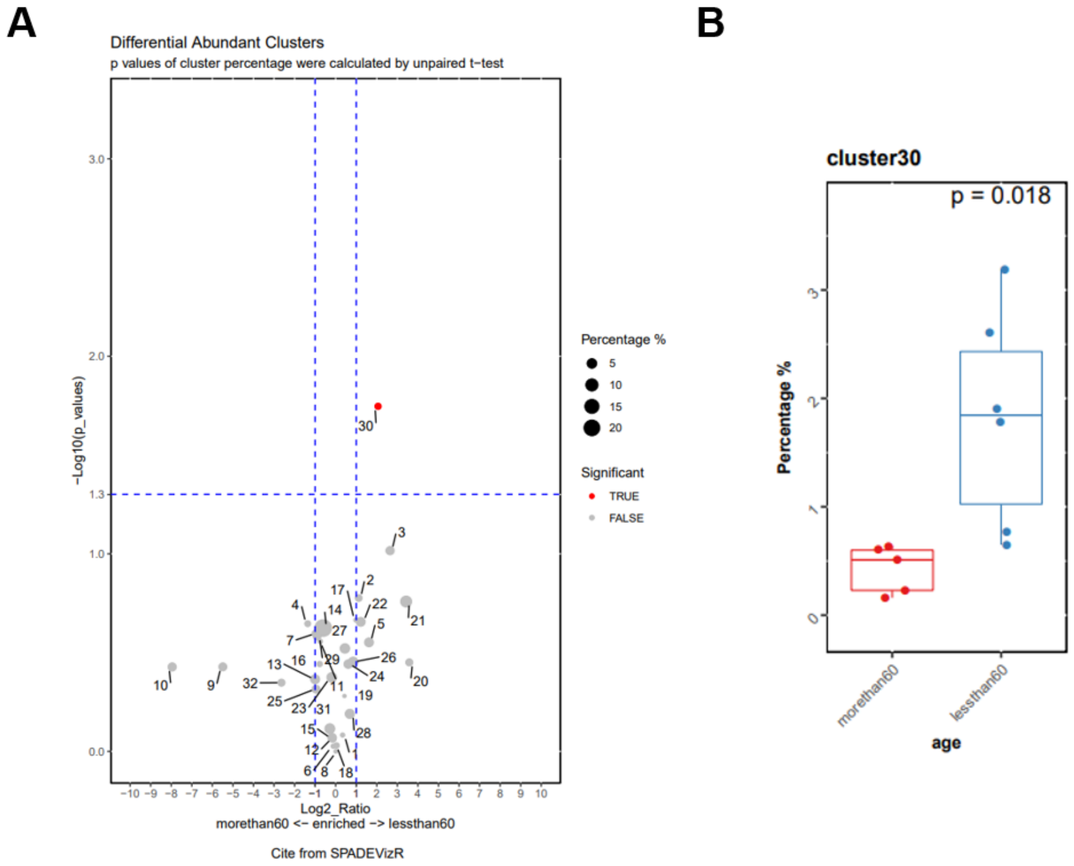

Supplementary Figure S9. A. Volcano plots reflecting the pairwise differences in the abundance of cell subsets among the two groups of PBMCs, p values of cluster percentage were calculated by unpaired t-test. **B.** Box plot displays immune cell subsets with significant differences over clinical characteristics of DHL patients in different age groups.

The cellular subsets of patients in two age groups (below/above 60) were further analyzed. The results show that the abundance of Cluster 30 was significantly different between the two groups. Cluster 30 is a $CD8^+$ naïve T cell with characteristics of $CD45RA^+$ and $CCR7^+$ $CD8^+$ T cells. Furthermore, the box plot boxplot shows that naïve $CD8^+$ T cells from patients over 60 years old significantly decreased, which is consistent with previous study (Immunol Cell Biol. **2022**, 100(10):805-821.).
